# Comparison of The Efficacy and Safety of Palomo, Ivanissevich and Laparoscopic Varicocelectomy in Iranian Infertile Men with Palpable Varicocele

**DOI:** 10.22074/ijfs.2018.5158

**Published:** 2018-01-15

**Authors:** Kamal Hosseini, Masoumeh Nejatifar, Ali Kabir

**Affiliations:** 1 Department of Urology, School of Medicine, Iran University of Medical Sciences, Tehran, Iran; 2 Shahid Beheshti University of Medical Sciences, Faculty of Public Health, Tehran, Iran; 3 Minimally Invasive Surgery Research Center, Iran University of Medical Sciences, Tehran, Iran

**Keywords:** Infertility, Male, Semen Analysis, Varicocele

## Abstract

**Background:**

This study aimed to compare the effects of three commonly used varicocelectomy techniques namely, open retroperitoneal ligation (Palomo), open inguinal ligation (Ivanissevich) and laparoscopy, in Iranian infertile men.

**Materials and Methods:**

This retrospective study was conducted on 70 infertile men with palpable varicocele who underwent one of the varicocelectomy techniques namely, Palomo, Ivanissevich, or laparoscopy. Basic information about semen parameters were collected and registered prior to the surgery. Three months after the surgery, semen parameters and surgical complications were investigated in all patients.

**Results:**

The Palomo technique was significantly associated with fewer complications compared to other techniques (P=0.006). The means of sperm concentration, normal motility and normal morphology were significantly different among the three groups after surgery (P=0.025, 0.023 and 0.047, respectively). However, after adjustment for potential confounders, in addition to the baseline values of semen parameters, significant differences were observed only in sperm concentration among the groups (P=0.040).

**Conclusion:**

Varicocelectomy improved sperm parameters. The Ivanissevich technique was more effective in improving sperm concentration compared to the laparoscopic method. The lowest rates of complications were related to the Palomo technique.

## Introduction

Reproductive health is a global health priority and infertility is one of its critical components regarded as a global health concern (1). The prevalence of infertility in developed and developing countries is 3.5-16.7% and 7-9%, respectively (2). Although in many communities particularly in developing ones, women are usually held responsible for infertility and male infertility is widely ignored (3). Male factors account for half of the infertility cases (4). In Iran, the prevalence of infertility is 10.9% (10.6 and 2.7% for primary and secondary infertility, respectively). Male factor conditions (with a prevalence of 34%) are the most prevalent causes of infertility in Iranian couples (5). Varicocele is the most well-known reversible cause of male infertility (6).

About one-sixth, two-fifths, and four-fifths of general male population and in men with primary and secondary infertility are diagnosed with varicocele, respectively (7, 8). Amajority of varicoceles are unilateral left-sided (90%) (9). Nowadays, radiologic techniques (embolization or sclerotherapy), open surgical techniques for ligation of the spermatic vein (using inguinal, subinguinal, and retroperitoneal methods), microsurgery (using inguinal and subinguinal methods), and laparoscopic varicocelectomy are used for the treatment of varicocele (8). Recurrence and hydrocele are complications which are commonly reported after varicocele surgery (10). Different studies have reported controversial results regarding the effect of various varicocelectomy techniques on male infertility. Therefore, no agreement has been reached yet about the "gold standard" of varicocele treatment (6).

In many developing countries, including Iran, it is notpossible for young couples to use the assisted reproductivetechnology (ART) due to its economic burden; therefore, in these countries, it is required to seek more affordableeffective approaches that are associated with fewer side ef. fects (11). This study aimed to compare the effects of threecommonly used varicocelectomy techniques namely, openretroperitoneal ligation (Palomo), open inguinal ligation(Ivanissevich) and laparoscopy, in Iranian infertile men.

## Materials and Methods

This retrospective study was conducted on infertile men with confirmed varicocele. These men had multiple abnormal semen analysis results and a 3-5 year history of primary infertility even after different medical treatments. The subjects were selected from individuals who referred to Hazrat Rasoul-e-Akram medical center in Tehran, Iran between 2009 and 2015. Cases with a history of previous scrotal or inguinal operation were excluded from our study. Using G*Power 3.1.9.2 and considering equal number in each group and 0.386 for effect size from means of postoperative sperm concentration (million/mL), 0.05 for alpha, 0.80 for power, and 3 for number of groups, a total sample size of 70 patients was calculated (12).

Convenience sampling was done by an expert clinician. Then, patients’ medical history was recorded and physical examination (mode of presentation laterality and varicocele grade) and semen analysis were done for each subject. The results of the last semen analysis before surgery, were considered as the baseline. The patients were classified into three clinical groups: grade 1 (palpable only with a Valsalva maneuver), grade 2 (non-visible but palpable without a Valsalva maneuver), and grade 3 (palpable and visible).

The status of atrophy in patients before and after surgery was examined using scrotal Doppler ultrasonography. Atrophy was defined as a testicular volume of <16 ml. According to WHO definition, normal semen samples have a volume of =1.5 mL, a sperm concentration of =15 million per mL, motility (movement of the sperm) value of =32% with forward progression (sum of type A and type B), total motility (sum of type A, B and C) of =40% and =4% normal morphology (13).

The sperm concentration was measured using a haemocytometer utilizing a neubauer sperm counting chamber after immobilization of spermatozoa by neutral formalin. The sperm motility was assessed by scanning a few fields under high-dry objective, until a total of =200 spermatozoa was enrolled and the sperm morphology was assessed on the basis of differential counts of morphologically normal and abnormal spermatozoa sorts on Pap-stained slides. Different types of sperm motility were classified into four groups: Quick dynamic (type A), Slow dynamic (type B), Non-dynamic (type C), and Immotile (type D). Based on a previous study, motility was defined as the average percentage of forward progression (sum of type A and B) divided by all four types of motility (14).

Information about time of surgery (since anesthesia time), type of anesthesia, and level of pain after surgery were collected for all the patients. Pain was measured using a visual analogue scale (VAS) ranging from zero (no pain) to 10 (severe pain). Three months after the surgery, semen parameters and surgical complications (atrophy, hematoma, recurrence, hydrocele, pneumoscrotum, significant nausea and vomiting, infection, ileus, next organ damages as well as need for blood transfusion, re-operation, changing the laparoscopic surgery to open, and other conditions) were investigated in all patients via clinical examinations, ultrasound test, and semen analysis. An urologist who was completely blind to the medical history and semen analysis, carried out all physical examinations.

Taking into consideration the clinical indication and patients’ preference, they were allocated to different groups of varicocelectomy. All surgeries were done by a single urologist. To compare the effects of surgery in each group, we used Mann-Whitney, Wilcoxon, and McNemar’s tests. To compare the effects of the type of surgery on semen parameters and to compare their side effects, we used chi- square or Kruskal-Wallis test, as appropriate.

Also, univariate general linear model was utilized to compare the effects of the type of surgery on semen parameters, by considering the baseline values of semen parameters as covariates and controlling the effect of other potential confounders. For sensitivity analysis, an additional univariate general linear model was used by controlling other residual (or potential) confounders that were not different among the three groups.

In order to predict the effect of varicocelectomy on semen parameters, we used linear regression in a stepwise manner. In this study, the level of statistical significance was set as a P value less than 0.05. All analyses were performed using SPSS 20 software (SPSS Inc., Chicago, Illinois, USA). All surgeries performed in this study were in accordance with the institutional ethical standards and the study protocol was confirmed by Ethics Committee of Iran University of Medical Sciences, Tehran, Iran. Written informed consent was signed by all participants.

## Results

There were 25, 23 and 22 cases in Palomo, Ivanissevich and laparoscopic groups with the mean age of 25.97 ± 5.7 years old which was not significantly different among the groups (P=0.352). In 76% of subjects, varicocele was in left side with no statistically significant differences among the three groups (P=0.513). Grade 3, 2 and 1 of varicocele were observed in 67, 30 and 3%, respectively. The rate of varicocele grade 3 in the Palomo group was significantly higher than that of laparoscopic (P=0.005) and Ivanissevich (P=0.047) groups. Sperm concentration was abnormal in 30 subjects accounting for 42.85% of patients population; there were no significant differences in this parameter among the three groups (P=0.138). 

Moreover, there were no statistically significant differences in other parameters of semen analyzed before the surgery, among the three groups. There was no atrophy before the surgery in 56% of the patients (n=39). Presence of atrophy significantly varied among different groups (64, 35 and 32% of cases in Palomo, Ivanissevich and laparoscopic groups, respectively; P=0.046). There was a significant difference among the three groups in terms of the mean duration of surgery (longer in laparoscopic type than two others) and type of anesthesia (general anesthesia in most cases of laparoscopic type and spinal anesthesia in the other methods) (P<0.001 for both comparisons) (Table 1).

The results showed that after surgery, the Palomo technique was significantly associated with fewer complications compared to other techniques (12, 55 and 44% for the Palomo, laparoscopic and Ivanissevich groups, respectively, P=0.006). In all group, no one had significant nausea and vomiting, infection, ileus, and next organ damages specifically intestinal damage as well as need for blood transfusion, re-operation, and changing the laparoscopic surgery to open method. In general, the most common complications were hydrocele in 21.4% (n=15), recurrence in 10% (n=7), and hematoma and pneumoscrotum each in 8.60% (n=6) of the patients. Pain after surgery was similar among all groups (Table 2).

Post-surgery semen analysis of all 70 subjects showed decreases in sperm concentration, normal motility and normal morphology in 7 (10%), 5 (7.4%) and 4 patients (5.9%), respectively. In these patients with semen parameters worsened after the surgery (n=13), from 5 patients with atrophy at baseline, only one had atrophy after the surgery and recurrence was observed in three of them. Moreover, 30 patients had abnormal sperm concentration and 67 patients had abnormal sperm motility at baseline; following the surgery, sperm concentration and sperm motility were within the normal range in 73.3% (n=22) and 40.2% (n=27) of these individuals, respectively (P<0.001 for both). 

**Table 1:** Basic characteristics of the patients in different groups of surgical treatment


Item	Group			P value	Power (%)
	Laparoscopyn=22	Ivanissevichn=23	Palomon=25				

Age (Y)^**, ***^	26.59 ± 6.05	26.78 ± 6.01	24.68 ± 5.23	0.352	64.01
Sperm concentration (million/mL)^**, ***^	13.09 ± 9.88	18.26 ± 13.38	14.96 ± 12.89	0.262	67.31
Sperm normal motility (%)^**, ***, #^	16.86 ± 6.77	19.56 ± 8.51	18.72 ± 9.90	0.639	59.32
Sperm normal morphology (%)^**, ***^	46.59 ± 14	48.04 ± 12.13	47.17 ± 15.87	0.871	51.04
Atrophy, n(%)^&^					
Positive	7(32)	8(35)	16 (64)	0.046	
Negative	15(68)	15(65)	9(36)		
Mode of presentation laterality, n(%)^&^					
Left unilateral	18(82)	18(78)	17(68)	0.513	16.31
Bilateral	4(18)	5(22)	8(32)		
Varicocele grade, n(%)^***^					
I	1(5)^a*^	1(4)^a^	0(0.00)	0.020	
II	11(50)^a^	7(31)^a^	3(12)		
III	10(45)^a^	15(65)^a^	22(88)		
Duration of surgery^**, ***^	61.59 ± 9.43	49.35 ± 6.08^b^	51.60 ± 8.50^b^	<0.001	
Type of anesthesia, n(%)^&^					
General	20(91)	5(22)	8(32)	<0.001	
Spinal	2(9)	18(78)	17(68)		


All variables refer to the condition of patients before surgery, ^**^; Values are presented as mean ± SD, ^***^; The comparisons were made by Kruskal-Wallis test, ^#^; Normal motility is sum of A+B motility type, and ^&^; The comparisons were made by Chi-Square test. The same lowercases showed no significant differences in the post-hoc Mann-Whitney tests.

**Table 2:** The occurrence of postoperative complications in different groups


Item	Group Total				P value	Power (%)
		Laparoscopyn=22	Ivanissevichn=23	Palomon=25			

Complications^*^	12(55)	10(44)	3(12)	25	0.006	
Hematoma^**^	1(4.5)	4(17)	1(4)	6(8.60)	0.657	51.23
Recurrence^**^	5(23)	2(9)	0(0.00)	7(10)	0.028	
Hydrocele^**^	8(36)	5(22)	2(8)	15(21.40)	0.059	48.49
Pneumoscrotum^**^	6(27)	0(0.00)	0(0.00)	6(8.60)	0.001	
Pain^***^	2.19 ± 1.40	2.09 ± 1.64	2.71 ± 1.87		0.430	59.79


Data are presented as mean + SD or n (%). ^*^; The comparisons were made by Chi-Square test. Complication: refers to any adverse effect observed in everybody, ^**^; The comparisons were made by Fisher’s exact test between the laparoscopic and open surgical techniques, and ^***^; The comparisons were made by Kruskal-Wallis test.

Mean values of semen parameters after surgery indicated significant improvements in all groups of varicocelectomy. The results of the univariate general linear model (by considering the preoperative values of semen parameters as covariates) revealed that the means of sperm concentration, normal motility and normal morphology were significantly different among the three groups after the surgery (P=0.025, 0.023 and 0.047, respectively). Mean values of sperm concentration and normal motility in the patients in Ivanissevich and Palomo groups were better than those of patients in laparoscopic group; however, Palomo technique had significantly better effect on normal morphology only compared to the laparoscopic technique (Table 3).

Comparing the mean differences of semen parameters among the three groups of varicocelectomy confirmed the results of univariate general linear model. We also used a univariate general linear model for controlling other factors (i.e. duration of surgery, atrophy before surgery, type of anesthesia and grade of varicocele) which were different among the three groups. The results of this analysis showed a significant difference among the groups just in terms of sperm concentration (P=0.040). Post-hoc analysis revealed that this difference was statistically significant only when comparing Ivanissevich (15.13 ± 8.69 million/mL) and laparoscopic (8.77 ± 8.94 million/mL) groups (P=0.008) (Table 4).

Age distribution and mode of presentation laterality was not significantly different in the three groups. Nonetheless, the power of this study to detect differences was low. Thus, we can consider the effect of these variables as residual confounders. Controlling these variables in an additional univariate general linear model showed that there was no significant differences among the three groups in terms of improving all semen parameters.

**Table 3:** Comparison of the results of surgery before and after utilization of three varicocelectomy techniques


Item	Group			P value	Power (%)
	Laparoscopyn=22	Ivanissevichn=23	Palomon=25		

Sperm concentration, million/mL^*^					
BS^**^	13.09 ± 9.88	18.26 ± 13.38	14.96 ± 12.89		
AS^***^	21.86 ± 10.28	33.39 ± 14.66^a^	29 ± 13.69^a^	0.0255^&^	
P value^#^	0.001	<0.001	<0.001		
Sperm normal motility (%)^*, ^^					
BS	16.86 ± 6.77	19.56 ± 8.51	18.72 ± 9.90		
AS	23.81 ± 9.55	31.95 ± 13.12^b^	32.80 ± 12.99^b^	0.0235^&^	
P value^#^	0.004	<0.001	<0.001		
Sperm normal morphology (%)^*^					
BS	46.59 ± 14	48.04 ± 12.13	43.40 ± 20.03		
AS	54.55 ± 12.71^c^	57.39 ± 10.32^cd^	58.40 ± 15.72^d^	0.0475^&^	
P value^#^	<0.001	0.001	<0.001		
Atrophy, n(%)^£^	0(0.00)	3(14.29)	1(4.55)	0.294	4.26


BS; Before surgery, AS; After surgery, ^*^; Values are presented as mean ± SD, ^**^; BS refer to values before surgery, ^***^; AS refer to values after surgery, ^#^; The comparisons were made by Wilcoxon test, ^&^; The Univariate general linear model was used for comparisons among the three groups, by considering the preoperative values of semen parameters as covariate, ^^^; Normal motility is sum of (A+B) motility type, ^£^; The comparisons were made by Fisher’s exact test between the Laparoscopic and the open surgical techniques. The same lowercases showed no significant differences in post-hoc tests.

**Table 4:** Comparing the mean differences of indices before and after surgery among patients undergoing three different surgical techniques


Item	Group			P value
	Laparoscopyn=22	Ivanissevichn=23	Palomon=25	

Sperm concentration (million/mL)^***^	8.77 ± 8.94^a^	15.13 ± 8.69^b^	14.04 ± 11.51^ab^	0.023
Sperm normal motility (%)^#, &^	6.95 ± 9.11	12.39 ± 9.87^c^	14.08 ± 8^c^	0.014
Sperm normal morphology (%)^^^	7.95 ± 4.27^d^	9.34 ± 10.47^de^	15 ± 12.4^e^	0.019


^*^; All values are presented as mean ± SD. All comparisons were made by Kruskal-Wallis test. ^***^; Mean count after surgery-mean count before surgery, ^#^; Mean normal motility after surgery-mean normal motility before surgery, and normal motility is sum of (A+B) motility type, ^^^; Mean normal morphology after surgery-mean normal morphology before surgery. The same lowercases showed no significant differences in post-hoc tests.

**Table 5:** Stepwise linear regression model for indices of semen analysis after the surgery


Dependent variable	Independent variable	Unstandardized coefficients		Standardized beta	P value	Model	
		Beta	SE of beta			R square	P value

Sperm concentration after surgery	Sperm concentration before surgery	0.761	0.105	0.678	<0.001	0.531	<0.001
	Laparoscopic surgical treatment	-7.587	2.917	-0.243	0.012		
	Atrophy before surgery	-5.449	2.614	-0.196	0.042		
Normal motility of sperm after surgery	Sperm normal motility before surgery	0.992	0.146	0.649	<0.001	0.505	<0.001
	Laparoscopic surgical treatment	-6.334	2.637	-0.229	0.020		
Normal morphology of sperm after surgery	Sperm normal morphology before surgery	0.535	0.070	0.629	<0.001	0.608	<0.001
	Sperm normal motility before surgery	0.354	0.123	0.241	0.005		
	Palomo surgical treatment	5.375	1.953	0.217	0.008		


Varicocelectomy helps to improve atrophy (P<0.001). So, at all ages and all surgery groups, among 31 patients who had atrophy at baseline, improvement in this respect was seen in nearly all of them (n=26, 83.9%), except for 2 patients at the age of 24 and 36. Atrophy was unknown for 3 patients. Moreover, except for the patients in Ivanissevich group, this positive effect was confirmed in patients of the other groups (P=0.016 for laparoscopy and P<0.001 for Palomo).

The results of stepwise linear regression showed that sperm concentration prior to the surgery, laparoscopic varicocelectomy, and atrophy prior to the surgery were the prognostic factors that could significantly predict the sperm concentration after the surgery. Laparoscopic varicocelectomy and presence of atrophy before the surgery have a negative impact on sperm concentration after the surgery. The values of normal motility before surgery and laparoscopic varicocelectomy were independent factors for predicting the normal motility after surgery. In addition, the number of sperms with normal morphology after the surgery, depends on the values of normal morphology before surgery and normal motility, as well as the utilization of Palomo technique. The adjusted R-square of the models (0.531, 0.505, and 0.608) indicates the higher accuracy of regression models in predicting the morphology and concentration, regardless of the number of independent variables entered the model. In each of the models, higher standardized beta indicates higher values of a variable in predicting the dependent variable. The values of each semen parameter prior to the surgery (e.g. sperm concentration) had the highest values in prediction of these parameters (e.g. sperm concentration) after the surgery (Table 5).

## Discussion

In this study, following the surgery, sperm concentration, normal motility, and normal morphology worsened in 10, 7.4, and 5.9% of patients, respectively. Based on univariate analysis, sperm concentration, normal motility and normal morphology after surgery using Ivanissevich and Palomo techniques, were better than those of laparoscopic group; but after controlling for confounders, a significant difference was seen only between Ivanissevich and laparoscopic techniques.

A similar study on 100 infertile patients who underwent varicocelectomy, showed a significant difference between open inguinal or laparoscopy methods in terms of sperm concentration and motility (15). A quasi-experimental study comparing open inguinal and laparoscopic Palomo in 50 patients, reported no significant differences between them in terms of sperm concentration and morphology, three and six months after the surgery (10).

According to other studies that had no controlling for confounding factors and did not consider the type of varicocelectomy, varicocelectomy could lead to significant improvements in sperm concentration, motility, and morphology. The results of our study are in line with the mentioned studies and confirm their findings (16, 17). Obviously, good quality and quantity of sperm before the surgery lead to better surgical outcome for the majority of patients. However, in this study we sought to find out which varicocelectomy technique has an additive effect in improving semen parameters. As regression models showed, laparoscopy had an inverse relationship with sperm concentration and motility after the surgery. In addition, these models showed that Palomo varicocelectomy was able to improve the mean normal morphology after the surgery.

The results of this study showed that laparoscopic and Palomo surgery had a positive effect on the improvement of the atrophy. Regarding Ivanissevich technique, at least in short-term follow-up in this study, no improvement in atrophy was seen; however, at least in terms of sperm concentration, changes were in direction to improve.

Nevertheless, to check the efficacy of varicocelectomy, we need to know post-surgery fertility status which was not assessed in this study. The best modality for treatment of varicocele in infertile men is a modality which highly improves semen and increases pregnancy rates with minimum complication rates (recurrence, hydrocele, and atrophy). Thus, an ideal technique not only preserves the lymph nodes and spermatic vessels, but also closes all external and internal spermatic veins. Although so far, no treatment modality has been introduced as a "gold standard" of varicocele treatment. According to the literature, compared to other varicocelectomy techniques, microscopic varicocelectomy (MV), despite its need for more operative time, surgical skills and experiences, was accepted as a standard treatment which had the lowest postoperative recurrence and complication rates (4). The findings of our study showed no significant differences among the three types of varicocelectomy in terms of complications after the surgery, which can be attributed to the method of our study (which consisted of non-random sampling, small sample size, and short follow-up period). It might be also due to the real low incidence of these complications in similar patients.

Overall rate of complications in open varicocelectomy has been reported to be slightly higher than laparoscopic varicocelectomy (8 vs. 6%, respectively) but this difference is not significant. Recurrent symptoms of varicocele were observed only in five and two patients in laparoscopic and Ivanissevich group, respectively. Other studies have also shown that higher grades of preoperative varicocele lead to increased risk of recurrence that can be secondary to multiple collateral venous channels (15). This can be applicable to our study as well because the majority of the patients in both groups were patients with varicocele grade 3. Another study has also reported the high rate of recurrence in laparoscopic surgery (10, 18-20).

Hydrocele after the surgery has an incidence rate of up to 10% of cases regardless of type of varicocelectomy (21). In the present study, hydrocele was observed in eight, five and two patients in laparoscopy, Ivanissevich, and Palomo group, respectively. Some studies have reported lower incidence of hydrocele in open inguinal group than laparoscopy group (10, 18, 19); but, some others have reported completely opposite results (15, 21, 22). A meta-analysis showed that the incidence of hydrocele is 8.24% after Palomo surgery, 2.84% after laparoscopic surgery, and 7.3% after macroscopic inguinal (Ivanissevich) or subinguinal varicocelectomy (21). Another study indicated that the recurrence rate and incidence of hydrocele were higher in patients undergoing Palomo surgery than those undergoing inguinal microsurgical procedure (22). This controversy could be due to an inadequate follow- up period in this study because most cases of hydrocele occur nine months after varicocelectomy (15).

There are some limitations in our study such as the retrospective nature of study, non-random sampling, small sample size, short follow-up period and no hormonal and fertility assessment. Moreover, there is a risk of selection bias. A higher proportion of men who underwent a Palomo repair had bilateral disease. Similarly, those who underwent this procedure had a higher grade of varicocele and higher incidence of atrophy, as defined in the study. This might have an impact on the results. However, we used a general linear model in our analyses to overcome this problem and fix this bias. Performing all surgeries by a single surgeon may cause dependency of the results to the surgeon; although, it removed inter-observer bias. In real scenario, most cases are like our cases with high grade of varicocele. So, our results are relatively generalizable.

## Conclusion

Varicocelectomy improves sperm parameters. Palomo, Ivanissevich, and laparoscopy methods were similar in terms of sperm normal motility and morphology. However, Ivanissevich was more effective in improving sperm concentration. Regarding complications, Palomo technique caused the lowest rate of post-surgery complications. It seems necessary to conduct further studies with longer follow-up periods to clarify the effect of different types of varicocelectomy on semen parameters and pregnancy rate in Iran.
